# Modelling Furrow Irrigation-Induced Erosion on a Sandy Loam Soil in Samaru, Northern Nigeria

**DOI:** 10.1155/2014/982136

**Published:** 2014-11-11

**Authors:** Jibrin M. Dibal, H. E. Igbadun, A. A. Ramalan, O. J. Mudiare

**Affiliations:** ^1^Department of Agricultural and Environmental Resources Engineering, Faculty of Engineering, University of Maiduguri, PMB 1069, Maiduguri, Borno State, Nigeria; ^2^Department of Agricultural Engineering, Faculty of Engineering, Ahmadu Bello University, Zaria, Nigeria

## Abstract

Assessment of soil erosion and sediment yield in furrow irrigation is limited in Samaru-Zaria. Data was collected in 2009 and 2010 and was used to develop a dimensionless model for predicting furrow irrigation-induced erosion (FIIE) using the dimensional analyses approach considering stream size, furrow length, furrow width, soil infiltration rate, hydraulic shear stress, soil erodibility, and time flow of water in the furrows as the building components. One liter of water-sediment samples was collected from the furrows during irrigations from which sediment concentrations and soil erosion per furrow were calculated. Stream sizes *Q* (2.5, 1.5, and 0.5 l/s), furrow lengths *X* (90 and 45 m), and furrow widths *W* (0.75 and 0.9 m) constituted the experimental factors randomized in a split plot design with four replications. Water flow into and out of the furrows was measured using cutthroat flumes. The model produced reasonable predictions relative to field measurements with coefficient of determination *R* ^2^ in the neighborhood of 0.8, model prediction efficiency NSE (0.7000), high index of agreement (0.9408), and low coefficient of variability (0.4121). The model is most sensitive to water stream size. The variables in the model are easily measurable; this makes it better and easily adoptable.

## 1. Introduction

Irrigation has been recognized to have been playing a crucial role in addressing the central challenges caused by food insecurity and rainfall uncertainty. It offers more yield assurance than rainfed agriculture and tends to improve the quality and value of crop yields. It is also often the key to successful commercial production of certain crops that cannot tolerate water stress or require very close regulation of inputs [1]. Furrow irrigation is especially recommended for growing row crops on medium to heavy textured soils. It is preferred over other surface irrigation methods due to its simplicity and low capital cost [2]. In Samaru, northern Nigeria, furrow irrigation is one of the most widely used means of water application to crops. Furrow irrigation method has been understood as one of the common farming practices that causes soil erosion in the irrigated farms. Soil erosion impacts negatively both on the environment and on crop productivity. Sojka et al. [1] report 75% of Idaho furrow irrigated fields lost entire “*A*” horizon in the upper reaches together with a 2- to 4-fold increase in “topsoil” at the lower ends, reducing productivity by 25% over preerosion values and reducing yields by 20–50% in areas where topsoil is lost. Furrow irrigation-induced erosion (FIIE) has been identified as one of the greatest global threats to sustainable agricultural productivity and to clean water. Preventing irrigation-induced erosion from irrigated agriculture is therefore imperative to the preservation of natural ecosystems [1]. Data on FIIE needed for planning and management of furrow irrigation is scarce in the study area. Modelling can be an effective means of predicting and planning against FIIE. Reasonably tested FIIE models are needed to estimate soil erosion in furrow irrigated fields from changing irrigation practices or to allocate soil erosion limits for various farming/management practices.

In Nigeria, up to 90% of irrigated farms are surface-irrigated, out of which furrow irrigation is one of the most widely practiced methods [3, 4]. But serious soil erosion occurs during irrigation and it is more pronounced in furrow irrigation methods [1, 5]. Koluvec et al. [6] reported that 21% of the 15 million hectares of irrigated land in the United States of America (USA) is affected by soil erosion. Significant erosion can reduce crop productivity in fields and degrades water quality of receiving water bodies [7]. Carter [8] reported a 25% decrease in crop yields in southern Idaho due to furrow irrigation. Records on furrow irrigation erosion in Nigeria are hard to come by. The need for information on furrow irrigation erosion presses harder in the face of the high degree of unskillful handling of irrigation practices among many Nigerian farmers [9].

Typical furrow irrigation in Nigeria could be described as haphazard as the selection of the flow stream sizes, length of furrows, furrow widths, depth of tillage, choice of direction of flow of water, frequency of water application, cropping pattern, and so forth does not follow specific pattern, determinants, or schedule. Mostly, the length of the farms determines the lengths of furrows, or it is irregularly subdivided into smaller lengths, sometimes as short as 20 m; that can be filled up quickly during irrigation to shorten the duration of their water. Consequently, a lot of soil might have been lost during furrow irrigation in Nigeria. However, the quantity of soil loss taking place on Nigerian farms due to furrow irrigation is still unknown, especially with reference to particular flow stream size, furrow length, and furrow width. Reliable quantitative data on the extent and rates of soil erosion is necessary for sustainable and comprehensive assessment of the magnitude of the problem for developing effective soil conservation measures. Another gap that exists in the present knowledge of soil loss, at least in this study area, is that no technique for estimating soil loss has been applied or tested in irrigated furrows despite its importance in soil loss studies. This implies that each time current soil loss data is required for planning and research in the study area, field estimations, data collections, laboratory analysis, and labour will have to be involved.

Given the continued growth of irrigation activities in Nigeria in response to the growing population and national support for irrigated agriculture, such as the case of* fadama* project amongst others, and the elevated national priority given to environmental and water quality protection, which is strongly linked to erosion, soil erosion in irrigated farms then becomes a major problem. But there is still relatively little or no published data that systematically quantifies the extent of irrigation-induced erosion. This is amazingly true despite many organized efforts from the government, nongovernmental organizations, and the academia toward funding for erosion inventory and for development of technology to understand, predict, and/or mitigate soil erosion that has been focused on rainfall-induced erosion only.

Having this problem at hand, the need for estimating soil erosion in furrow irrigated fields with a view to reducing it to the barest possible has therefore become very important. Thus, accurately simulating furrow irrigation erosion becomes practically indispensable for planning and evaluating management practices and for meeting water quality standards. Models are very important tools for understanding and predicting soil erosion and could be used in conservation planning and erosion control.

### 1.1. Furrow Irrigation-Induced Erosion Models

A variety of erosion models exist focusing on different spatial and temporal scales, with varying degrees of complexity and precision to address furrow irrigation-induced erosion. For example, Adeniji [10] developed equations for stream front advance distance (m), stream erosion rate (m/s), and water runoff (l/s) for furrow irrigation:

(1) $$\begin{array}{c} E=\left[A_{3}{\left(\frac{K_{S}}{tX_{\max}}\right)}^{B_{3}}\right]K_{6}, \end{array}$$

where  *E* = soil erosion rate (m/s) by a given furrow irrigation stream at the lower end of the length, *X*_max⁡_; *A*_3_ = function of stream size and soil aggregate stability; *K*_5_ and *K*_6_ = functions of acceleration due to gravity, mass density of water, and dynamic viscosity; and *B*_3_ = function of initial silt and clay content and soil dispersibility factor.

One of the limitations of (1) is that it did not include some variables such as soil infiltration rate, duration of water flow in the furrows, and the effect of furrow geometry. It could not therefore adequately address soil erosion in irrigated furrows. Further, besides the fact that the equation was developed for Saskatoon environment, the range of values of some factors such as *A*_3_ and *B*_3_ was not available and could vary in space. The model therefore could not give a straightforward estimate of soil erosion in irrigated furrow.

Gardner and Lauritzen [11] proposed an equation relating critical flow and critical slope in furrow irrigation that was amended by Criddle [12], Hamad and Stringham [13], Trout and Neibling [14], and Spofford and Koluvek [7]. Their equation all correlated soil erosion to nonerosive stream size and slope. But FIIE is a function of many other factors that were not captured in their equation.

Another two soil erosion models recently tested for uses in irrigation are water erosion prediction project (WEPP) [15] and the surface irrigation model (SRFR) [16]. WEPP model is a steady-state erosion model; erodibility parameters cannot change during irrigation. Preliminary evaluation showed the model did not predict any soil erosion unless default baseline erosion parameters were reduced [17]. The model overpredicted soil loss and deposition and only predicted when runoff was greatly underpredicted or detachment was greatly overpredicted. These factors indicate that the WEPP model overpredicts transport capacity in irrigation furrows.

The SRFR [16] is a surface irrigation model that simulates water advance, infiltration, and recession. It has a provision for the user to input furrow geometry, soil infiltration and roughness characteristics, and irrigation management. Some of the model output parameters are runoff, infiltration, irrigation efficiency, distribution uniformity, and deep percolation.

This model is not a steady-state erosion model like the WEPP model, so the erosion parameters can vary during irrigation. However, the model only predicts erosion from one furrow during a single irrigation. It also does not calculate the effects of tillage on soil erosion parameters or predict erosion from a field or watershed for several years. The advantage of the SRFR model is the more detailed representation of furrow irrigation hydraulics and non-steady-state erosion predictions. The limitation of this model is its complexity due to many components used in its building, especially the transport capacity component; furthermore the erodibility factor of the model did not represent furrow irrigation erosion in its entirety, and unity was assigned in it during the testing of the model. This makes the model difficult to adopt [18].

The Idaho Natural Resources Conservation Service (NRCS) in Idaho developed surface irrigation soil loss model (SISL) to estimate soil loss from furrow irrigated fields [1]. It is a simple empirical model that uses a formula similar to the universal soil loss equation (USLE). An evaluation of SISL showed that the absolute differences between measured and predicted values were often large [19] and it does not take into account the variability of soil erosion with respect to the major variables affecting furrow irrigation. The applicability of this model in estimating soil erosion in irrigated furrow is thus questionable. Further, the solutions of most of the models are generally subject to computational difficulties and inaccuracies. This study was consequently conceived to build up a model for estimating soil erosion in furrow irrigation in the semiarid region of northern Nigeria.

## 2. Materials and Methods

### 2.1. Model Development

The furrow irrigation-induced erosion (FIIE) model was developed using dimensional analysis following the concept of Buckingham *π*-theorem considering the variables in Table 1.

However, the principles of the Buckingham *π*-theorem require that all the variables to be used in the dimensional analysis be theoretically independent. Some of the variables in Table 1 were therefore eliminated, to arrive at the decision variables (Table 2) used in the model development.

### 2.2. Determination of the Dimensionless Groups (*π*-Terms)

The *π*-terms were determined using the dimensional analysis approach.

#### 2.2.1. Determination of *π*_1_

Consider

(i) $$\begin{array}{c} \pi_{1}=f\left(Q,\tau ,K,E\right)=f{\left[Q\right]}^{a1}{\left[\tau \right]}^{b1}{\left[K\right]}^{c1}\left[E\right],\\ M^{0}L^{0}T^{0}={\left[L^{3}T^{-1}\right]}^{a1}{\left[ML^{-1}T^{-2}\right]}^{b1}{\left[T^{3}L^{-3}\right]}^{c1}\left[ML^{-2}T^{-1}\right], \end{array}$$

(ii) $$\begin{array}{c} M\text{:}\;0=b1+1, \end{array}$$

(iii) $$\begin{array}{c} b1=-1, \end{array}$$

(iv) $$\begin{array}{c} L\text{:}\;0=3a1-b1-3c1-2, \end{array}$$

(v) $$\begin{array}{c} T\text{:}\;0=-a1-2b1+3c1-1. \end{array}$$

Substituting −1 for *b* into (iv) and (v) yields

(2) $$\begin{array}{c} a1=0,\;\;c1=-\frac{1}{3}. \end{array}$$

Therefore *π*_1_ = *f*_1_(*Q* ^0^ *τ* ^−1^ *K* ^−1/3^ *E*), or 
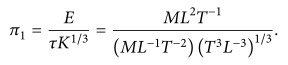
(vi) The above procedure was used to develop four other *π*-terms.

#### 2.2.2. Summary of the *π*-Terms

Consider

(3)  π1=EτK1/3, π2=XQ1/2K1/6, π3=WQ1/2K1/6, π3=K1/3I, π5=TQ1/2K1/2.

### 2.3. Combination of *π*-Terms

The *π*-terms obtained above were combined to form three *π*-terms; thus, a new *π*_1_ herein called *π*_1*n*_ was obtained by taking the product of *π*_1_, *π*_2_, *π*_3_, and *π*_4_. The new *π*_2_ called *π*_2*n*_ was obtained from product of *π*_2_ and *π*_3_. The original *π*_5_ was taken as the new *π*_3_ now called *π*_3*n*_:

(4)  π1n=EXWIτQK1/3, π2n=XWQK1/3, π3n=TQ1/2K1/2.

Hence the three *P*_*i*_ terms required and the equation can be written as

(5) $$\begin{array}{c} \pi_{1}=F\left(\pi_{2},\pi_{3}\right). \end{array}$$

## 3. Field Experimentation

### 3.1. Study Area

Data necessary for the completion of the model development were collected during the 2009/2010 and 2010/2011 irrigation seasons at the Irrigation Research Field of the Institute for Agricultural Research (IAR) farm, Samaru-Zaria, along Zaria-Sokoto road (11°1′N, 7°38′E, on the altitude of 686 m above mean sea level). Samaru is situated within the Northern Guinea savanna zone of Nigeria. The field was ploughed, harrowed, and ridged at 0.75 m spacing thereby creating the furrows in which irrigation was conducted. The furrows were 90 m long.

### 3.2. Field Measurements

#### 3.2.1. Preirrigation Data

The basic infiltration rate of the soil was determined using the inflow-outflow method [20]. Slope of the field was determined in each of the two trials using the dumpy level surveying instrument. The hydraulic shear stress, *τ*, was obtained following the guidance of Schwab et al. [21]. The soil erodibility, *K*, was determined by adopting the universal soil loss equation (USLE) “*K*” equation as it is in Wall et al. [22]. The time of flow of water was measured directly from the field with a stopwatch.

#### 3.2.2. Irrigation/Erosion Related Data

Prior to commencement of irrigations, water-sediment collection stations were established 5 m before the end of each furrow. Flow of water in the furrows was measured using a cutthroat flume installed 5 m from entry upstream of each of the furrows for the measurement of inflows and at the tail end of the furrows. Water flowing out of the furrows was measured as runoff. One liter of water-sediment samples was collected at each of the established measurement points for determination of sediment concentrations. These samples were filtered into preweighed metal containers; the collected residues were oven-dried at 105°C over 24-hour period and reweighed in laboratory. The sediment concentrations (g/l) that were calculated from the dried residues and the runoff volumes were used to calculate soil erosion per furrow. Runoff volume was calculated as the product of the runoff discharge (l/s) (from the downstream flumes) and duration of runoff discharge. Soil erosion at the end of the furrows was calculated as the product of the sediment concentrations and runoff volumes divided by the wetted area. The wetted areas were calculated as the product of the top widths of flow of water and the lengths of the furrows [8, 23–25].

From the dimensional analysis the general solution of (5) which gives three dimensionless groups characterizing this phenomenon can therefore be written as

(6) $$\begin{array}{c} \phi =\left(\frac{EXWI}{\tau QK^{1/3}},\frac{XW}{QK^{1/3}},\frac{T}{Q^{1/2}K^{1/2}}\right),\\ \frac{EXWI}{\tau QK^{1/3}}=F\left(\frac{XW}{QK^{1/3}},\frac{T}{Q^{1/2}K^{1/2}}\right). \end{array}$$

**Figure 1:**
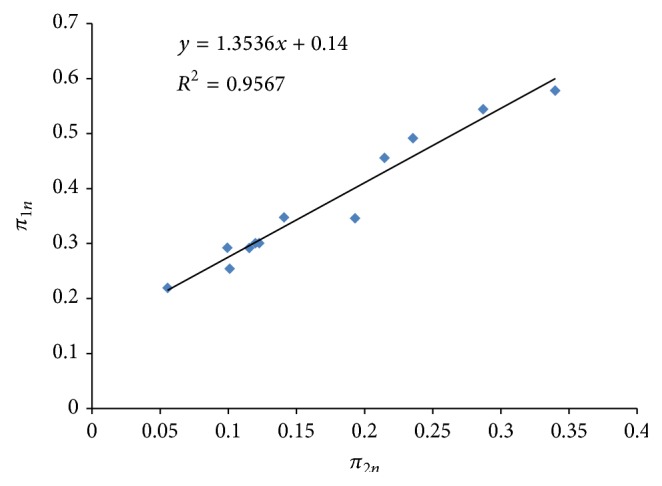
A plot of *π*_1*n*_ against *π*_2*n*_ (*π*_3*n* constant_).

**Figure 2:**
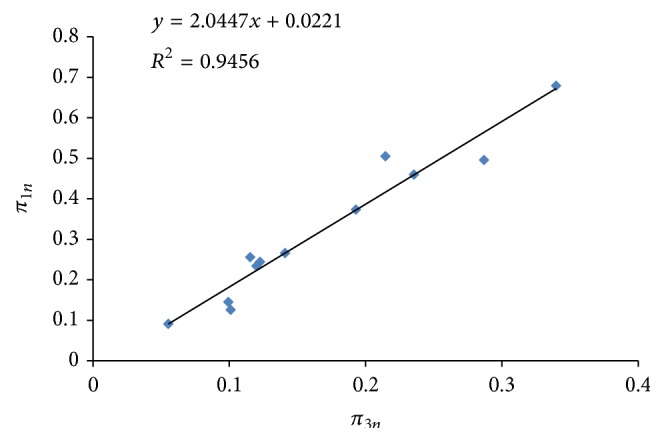
A plot of *π*_1*n*_ against *π*_3*n*_ (*π*_2*n* constant_).

Dimensionless plots of *π*_1*n*_ against *π*_2*n*_ and *π*_1*n*_ against *π*_3*n*_ were performed as shown in Figures 1 and 2. In all the cases, there exists good relationship between *π*_1*n*_ and *π*_2*n*_ and between *π*_1*n*_ and *π*_3*n*_ with coefficient of determination (*R* ^2^) in the neighborhood of 0.9500 as shown in Figures 1 and 2.

The regression equations derived from the graphs were used as the component equations in the model development:

(7)  π1n=0.1400+1.3536π2n R2=0.9567, π1n=−0.0221+2.0447π3n R2=0.9456.

It follows that *π*_1*n*_ can be obtained as linear combinations of (7).

That is,

(8) $$\begin{array}{c} \pi_{1n}=\gamma +\alpha \pi_{2n}+\beta \pi_{3n}. \end{array}$$

From (8), it can be deduced that

(9) $$\begin{array}{c} \sum y=n\gamma +\alpha \sum x_{1}+\beta \sum x_{2}. \end{array}$$

Multiplying through by *x*_1_ yields

(10) $$\begin{array}{c} \sum x_{1}y=\gamma \sum x_{1}+\alpha \sum x_{1}^{2}+\beta \sum x_{1}x_{2} \end{array}$$

and multiplying by *x*_2_ yields

(11) $$\begin{array}{c} \sum x_{2}y=\gamma \sum x_{2}+\alpha \sum x_{1}x_{2}+\beta \sum x_{2}^{2}. \end{array}$$

The values of *α*, *β*, and *γ* were obtained using the multiple linear regression analysis using the procedure detailed by Bernett et al. [26] and presented as follows:

(12) $$\begin{array}{c} \left[\begin{array}{ccc} n & \sum x_{1} & \sum x_{2}\\ \sum x_{1} & \sum x_{1}^{2} & \sum x_{1}x_{2}\\ \sum x_{2} & \sum x_{2}x_{1} & \sum x_{2}^{2} \end{array}\right]\left[\begin{array}{c} \gamma \\ \begin{array}{c} \alpha \end{array}\\ \begin{array}{c} \beta \end{array} \end{array}\right]\left[\begin{array}{c} \sum y\\ \begin{array}{c} \sum x_{1}y \end{array}\\ \begin{array}{c} \sum x_{2}y \end{array} \end{array}\right], \end{array}$$

where *y*, *x*_1_, and *x*_2_ represent *π*_1*n*_, *π*_2*n*_, and *π*_3*n*_, respectively, and *n* is number of observations, which is 12 in this case.

Substituting the values of *y*, *x*_1_, and *x*_2_ in (12) yielded

(13) $$\begin{array}{c} =\left[\begin{array}{ccc} 12 & 4.421 & 3.8752\\ 4.421 & 1.784 & 1.6527\\ 3.8752 & 1.6527 & 1.6087 \end{array}\right]\left[\begin{array}{c} \gamma \\ \alpha \\ \beta \end{array}\right]\left[\begin{array}{c} 2.0248\\ 0.8554\\ 0.8191 \end{array}\right]. \end{array}$$

Using Crammer's rule procedure, *γ*, *α*, and *β* were found to be −0.04618, 0.3973, and 0.2122, respectively.

Then (8) became

(14) $$\begin{array}{c} \pi_{1n}=-0.04618+0.3973\pi_{2n}+0.2122\pi_{3n}. \end{array}$$

### 3.3. Measure of Model Performance

To assess the extent of the accuracy of the simulation behavior of the model, the efficiency criteria (ECs) were used to study the error margin between the simulated and observed FIIE values. The ECs used were the coefficient of variation (CV), Nash-Sutcliffe efficiency (NSE), index of agreement (*d*), and root mean square error (RMSE) [27, 28]. The ECs were presented in

(15) $$\begin{array}{c} \left[\text{RMSE}=\sqrt{\frac{\sum_{i=1}^{n}{\left(O_{i}-P_{i}\right)}^{2}}{n}}\right],\\ \text{CV}=\frac{\text{S}.\text{D}}{\bar{O}},\\ d=1-\frac{\sum_{i-1}^{n}{\left(O_{i}-P_{i}\right)}^{2}}{\sum_{i=1}^{n}{\left(\left|P_{i}-\bar{O}\right|+\left|O_{i}-\bar{O}\right|\right)}^{2}},\\ \text{NSE}=1-\frac{\sum_{i=1}^{n}{\left(O_{i}-P_{i}\right)}^{2}}{\sum_{i=1}^{n}{\left(O_{i}-\bar{O}\right)}^{2}}, \end{array}$$

where *O* and *P* are observed and predicted FIIE values, respectively, and S.D and are $\bar{O}$ standard deviation and mean of observed values of FIIE.

## 4. Results and Discussion

### 4.1. The Prediction Equation

The general prediction equation for a system or process involving three *π*-terms formed by addition of the component equations as given in (14). Inserting the expressions of the *π*-terms yields the prediction equation as presented in

(16) EXWiτQK1/3=0.3973XWQK1/3+0.2122TQ1/2K1/2 −0.04618.

Equation (16) was further simplified to yield the prediction equation as presented in

(17) E=τQK1/3XWI ×0.3973XWQK1/3+0.2122TQ1/2K1/2−0.04618

or

(18) E=0.3973τI+0.2122TQ1/2τXWIK1/6 −0.04618τQK1/3XWI.

### 4.2. Model Validation

**Figure 3:**
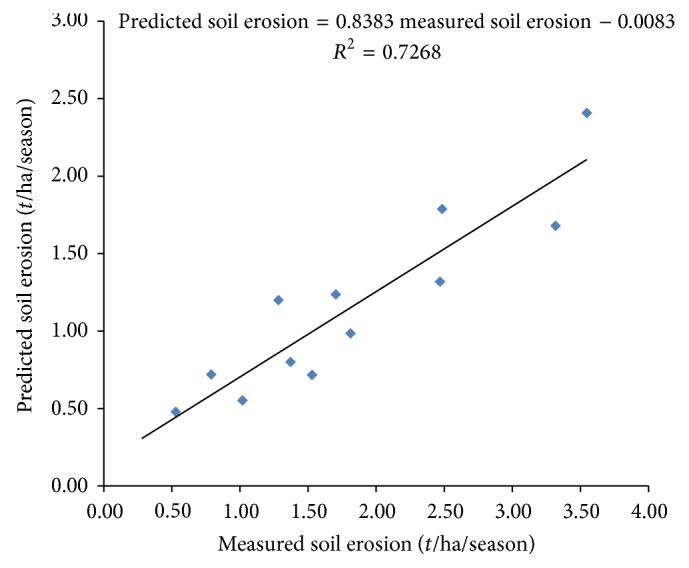
Comparison of predicted and measured soil erosion.

In order to assess the degree to which the developed model is an accurate representation of the real world, the soil erosion output simulated using the model (18) was validated against a different set of soil erosion data that were collected in trial 2. The relationship between the observed field data and model prediction is presented in Figure 3. The uncertainties in the model output and experimental outcomes produced the variation in the points on the figure. The slope and intercept of the regression equation being 0.8383 and 0.0083 were closer to unity and zero, respectively, and the *R* ^2^ of 0.7268 exhibits a high degree of agreement between the model output and the field measured soil erosion data. This implies that the model is a good representation of real soil erosion in irrigated furrow and demonstrates the model's ability to predict soil erosion in irrigated furrows. Any error in the model prediction is therefore expected to be within the confidence limit.

### 4.3. Measure of Model Performance

The performance indices of the model (18) are presented in Table 3. The index of agreement (*d*) indicated the best value, signifying the very good predictive capacity of the model [27]. Similarly, the value of the NSE falls within the range of 0.5 to 1; the model can then be judged to have a good performance. It was shown that the range of NSE lies between 1.0 (perfect fit) and −1 such that efficiency lower than zero would demonstrate a poor prediction capacity of the model [28].

The root mean square error (RMSE) and coefficient of variation (CV) were also low signifying low degree of variation between the observed and predicted values [27]. Generally, the analysis demonstrated a good predictive capacity of the model.

### 4.4. Sensitivity Analysis

In analyzing the sensitivity of the model to the building variables, the factor perturbation simulation (FPS) approach was adopted for its simplicity and accuracy [29, 30]. The sensitivity coefficients (S.C.) for each variable were obtained using

(19) $$\begin{array}{c} \text{S}.\text{C}.=\frac{\Delta E}{\Delta V}, \end{array}$$

where S.C. is the sensitivity coefficient and Δ*E* and Δ*V* are changes in furrow irrigation erosion and variables, respectively. During the analysis, one parameter was varied at a time while the other parameters were kept fixed to make sure that the sensitivity method was monocriteria. A 10% increase in the values of each was used in the analyses.

The result of the sensitivity analysis of the model is presented in Table 4. Perusal of the table shows that the sensitivity coefficients of the variables ranged from 128.03 to 0.0004 for all the independent variables. The results of the sensitivity analysis revealed that the model is most sensitive to changes in stream size that has the dominantly highest S.C. This could be attributed to its role in detachment and transportation of soil particles.

It was followed by soil erodibility, *K*, but with a very wide gap between them. This analysis also showed that the model is least sensitive to changes in time of flow. This implies that errors in measuring stream size may lead to larger errors in the model's prediction. Overall, the analysis emphasized the importance of stream size and hence the need to commit more attention and resources to its measurement.

## 5. Conclusions

Furrow irrigation is still one of the most used irrigation methods in Samaru-Zaria and environs. Furrow irrigation-induced erosion (FIIE) is one of the silent problems militating against sustainable irrigated agriculture in the area. Accurately simulating furrow irrigation erosion is essential for assessing loads and evaluating best management practices for meeting water quality standards. Irrigation-induced erosion has some unique differences from rainfall erosion, such as water flowing on to dry soil and furrow flow rate decreasing with distance and increasing with time. A model for estimation of furrow irrigation-induced erosion on a sandy loam soil has been developed. The fieldwork included measurements of flow and soil loss and runoff on experimental field plots. A close agreement was achieved between the model output and the practically measured soil erosion, demonstrating a high degree of confidence. The performance evaluation of the model shows that the model has high prediction efficiency signifying that the model has high accuracy and precision and can be used for schematization and watershed project. The sensitivity analysis using the factor perturbation approach revealed that the model is most sensitive to water flow stream size advocating for more attention to be paid to stream size during operation and management of furrow irrigation. Owing to possible variation in soil properties space and time and/or management practices, analysis of the seasonal variation of sensitivity of the model with respect to the building variables would facilitate better understanding of the performance of the model. Further studies are needed to extrapolate this study on a broader scale in Nigeria and across several soil types and testing in furrows longer than 100 m. This was not done here because of space constraint. Conducting this work on a long-term basis, on an institutional scale, say for 10 years, would improve on the reliability of soil erosion estimates in irrigated furrows.

## Figures and Tables

**Table 1:** Variables considered in the model development.

Variables	Symbols	Units	Dimensional symbols
Soil erosion	*E*	Kg/m^2^/yr	*ML* ^−2^ *T* ^−1^
Stream size	*Q*	m^3^/s	*L* ^−3^ *T* ^−1^
Slope	*S*	%	[ ]
Furrow lengths	*X*	m	*L*
Top widths of flow	*W*	m	*L*
Hydraulic radius	*R*	m	*L*
Infiltration rate	*I*	mm/hr	*LT* ^−1^
Time of flow	*T*	sec	*T*
Flow velocity	*V*	m/s	*LT* ^−1^
Manning's roughness coefficient	*n*	sec/m^1/3^	*TL* ^−1/3^
Acceleration due to gravity	*g*	m/s^2^	*LT* ^−2^
Density of water	*ρ*_*w*_,	kg/m^3^	*ML* ^−3^
Soil particle density	*ρs*,	kg/m^3^	*ML* ^−3^

**Table 2:** Decision variables and their corresponding dimensions.

Variables	Symbols	Units	Dimensional symbols (M, L, T)
Soil erosion	*E*	kg/m^2^/yr	*ML* ^−2^ *T* ^−1^
Stream size	*Q*	m^3^/s	*L* ^ 3^ *T* ^−1^
Furrow length	*X*	m	*L*
Top width of flow	*W*	m	*L*
Soil infiltration rate	*I*	mm/hr	*LT* ^−1^
Time of flow of water	*T*	s	*T*
Soil erodibility factor	*K*	Kg·hr/Nm^2^	*L* ^−3^ *T* ^ 3^
Hydraulic shear stress	*τ*	N/m^2^	*ML* ^−1^ *T* ^−2^

**Table 3:** Model performance.

Model performance indices	Value	Optimum value
CV	0.4121	0
NSE	0.7000	1
*D*	0.9408	1
RMSE	0.6428	0

**Table 4:** Results of sensitivity analysis.

Variables	Original values of variables	10% increase in values of variables	Original soil erosion value	Soil erosion response to 10% increase of values of variables	Change in soil erosion	Changes in variables' values	Sensitivity coefficient (S.C.)
Shear stress *τ*	25.783	28.361	0.981	1.080	−0.098	2.5783	−0.0381
Furrow length *X*	90	99	0.981	0.922	0.0600	9	0.00663
Top width of flow *W*	0.653	0.718	0.981	0.922	0.0600	0.0653	0.91415
Infiltration rate *I*	24.776	27.254	0.981	0.892	0.0900	2.4776	0.036
Stream size *Q*	0.0025	0.003	0.981	1.013	−0.032	0.00025	−128.03
Erodibility factor *K*	0.02	0.022	0.981	0.941	0.0404	0.002	20.1969
Time of flow *T*	481.8	529.98	0.981	1.003	−0.0212	48.18	−0.0004
